# Evaluation of Ecosystem Service Capacity Using the Integrated Ecosystem Services Index at Optimal Scale in Central Yunnan, China

**DOI:** 10.1002/ece3.71222

**Published:** 2025-04-11

**Authors:** Lanfang Liu, Jinliang Wang, Jie Li, Suling He, Yongcui Lan, Fang Liu

**Affiliations:** ^1^ Faculty of Geography Yunnan Normal University Kunming China; ^2^ Key Laboratory of Resources and Environmental Remote Sensing for Universities in Yunnan Kunming China; ^3^ Center for Geospatial Information Engineering and Technology of Yunnan Province Kunming China; ^4^ Southwest United Graduate School Kunming China

**Keywords:** driving force analysis, Integrated Ecosystem Service Index (IESI), InVEST model, scale effect, the Central Yunnan Province (CYP)

## Abstract

Understanding and quantifying the dynamic features of local ecosystem services (ESs) and integrating diverse ecosystem assessment results form crucial foundations for regional ES management. However, existing methods for integrating and objectively evaluating multiple ESs remain limited. Consequently, this research evaluates four key services based on the InVEST and RUSLE models in the Central Yunnan Province (CYP)—from 2000 to 2020: water yield (WY), carbon storage (CS), habitat quality (HQ), and soil conservation (SC). It then constructs an Integrated Ecosystem Service Index (IESI) using principal component analysis (PCA). Additionally, this study explores the factors driving the spatial divergence of ESs by employing the optimal parameter‐based geographical detector model (OPGD) at the optimal spatial scale. The results indicated that (1) the IESI was effectively applied in the CYP and could quantitatively and comprehensively integrate the assessment results of the four key ESs. (2) During the study period, the ESs in the CYP showed increasing trends for WY, HQ, and SC, while CS showed a decreasing trend. (3) The IESI during the study period exhibited a trend of initially decreasing and then increasing. The average IESI values for CYP were 0.7338 in 2000, 0.6981 in 2005, 0.6947 in 2010, 0.6650 in 2015, and 0.6992 in 2020. (4) A 4500 m × 4500 m grid was identified as the optimal spatial scale for detecting the spatial divergence of comprehensive ecosystem service (CES) in CYP, and relief degree of land surface (RDLS), slope, and the NDVI were the top three drivers based on q‐values. This study offers a more scientific and effective method for evaluating regional CES. It also provides a comprehensive analytical tool for balancing land use competition and assessing the effectiveness of policy implementation.

## Graphical abstract

The novelty of this research is to conduct a quantitative assessment of four key ESs—water yield (WY), carbon storage (CS), habitat quality (HQ), and soil conservation (SC)—in CYP from 2000 to 2020 and to propose an integrated method based on principal component analysis (PCA) to construct the Integrated Ecosystem Service Index (IESI).

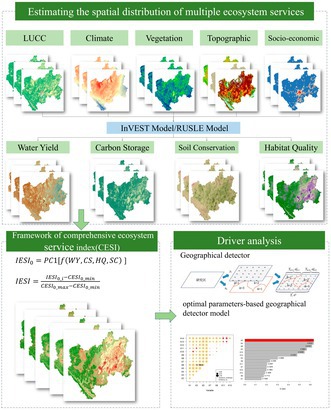

**Abbreviations**
CES: comprehensive ecosystem service
CS: carbon storage
CYP: Central Yunnan Province
ES: ecosystem service
ESV: ecosystem service value
HQ: habitat quality
IESI: Integrated Ecosystem Service Index
LUCC: Land Use/Cover Change
NDVI: Normalized Difference Vegetation Index
OPGD: optimal parameter‐based geographical detector model
PCA: Principal Component Analysis
RDLS: relief degree of land surface
RUSLE: Revised Universal Soil Loss Equation
SC: soil conservation
WY: water yield

## 1 Introduction

Ecosystem services (ESs) are the life‐support goods and services that are obtained directly or indirectly through the structure, function, and processes of ecosystems, encompassing the provisioning, regulating, and cultural services provided to humans, along with supporting services sustained for other services (Costanza et al. 1997; Kumar 2012). As human exploitation of natural resources has increased, human interference causes increasing pressure on an ecosystem's self‐regulatory capacity, leading to eco‐environmental degradation and a serious imbalance in ecosystem processes. The loss of ESs is irreversible and will seriously threaten regional and global ecological security (Millennium Ecosystem Assessment, 2005). Therefore, it is crucial to grasp the spatio‐temporal dynamics patterns and explore the driving mechanisms of ESs for addressing regional ES issues, maintaining ecological balance, and enhancing human well‐being.

In recent years, scholars have conducted numerous assessment studies on different ESs, ecosystems, and spatial–temporal scales. Regarding the study area, the current assessments mainly cover a wide range of geographic regions and ecosystem types, including countries (Hu et al. 2023a; Leh et al. 2013; Neugarten et al. 2020), densely populated cities and urban agglomerations (Elliot et al. 2022; Hysa et al. 2024; Wang et al. 2024), watersheds (Wang et al. 2021; Wanjala et al. 2018; Zhou et al. 2022), nature reserves (Gaylard et al. 2020; Mengist et al. 2023), and forest ecosystems (Nguyen et al. 2020), etc. These studies usually feature multiple scales, but the results of ES assessment in different types and regions are still difficult to compare. Additionally, Research on quantitative methods for key ESs is a crucial aspect of ES assessment, and the assessment pathways are mainly divided into two categories: economic methods and biophysical methods (Li 2019). Economic methods quantify the ecosystem service value (ESV) in monetary terms. Among these methods, the equivalence factor method is a primary approach for calculating ESV (Costanza et al. 1997; Xie et al. 2015), which is convenient to compute, as it assumes that the same ecosystem type has the same ESV equivalent. However, this method overlooks the spatio‐temporal heterogeneity of ES provision (Li 2019). Biophysical methods simulate biophysical quantities such as water yield (WY) by obtaining ecosystem observation data and then combining the data with relevant ecological models (Bagstad et al. 2013; Hu et al. 2015). These approaches objectively reflect the characteristics of ecosystem processes and the mechanisms of the formation of ES (Fu et al. 2013), making them more suitable for assessing ES characteristics under different LULC conditions at small scales (Fu and Zhang 2014). Current studies have focused on the quantitative assessment of ES based on either a single service (Kim et al. 2016; Lu et al. 2024) or multiple services (Sang et al. 2024; Wang et al. 2024). As environmental benefits continue to expand, there is an urgent need to explore new approaches for quantitatively integrating the assessment results of various ESs at the regional scale (Wu and Fan 2022). Constructing an Integrated Ecosystem Services Index (IESI) by integrating multiple ESs is the main research at present (Liu et al. 2024a). Currently, the main method for constructing IESI involves using cumulative equations (Lang and Song 2019; Wen et al. 2024) and the maximum value method, which assesses the relative highest importance among multiple ESs (Zhang et al. 2020). Additionally, some researchers have employed the analytic hierarchy process (AHP) to assign subjective weights to indicators for integrating multiple ESs (Liu et al. 2023a). However, most previous methods have not objectively considered the relative importance of indicators (Wu and Fan 2022). Therefore, there is a pressing demand for a new method to objectively and quantitatively construct IESI. Principal component analysis (PCA) shows promising results (Marsboom et al. 2018; Salata and Grillenzoni 2021). However, it has mainly been applied to reduce the number of bands while concentrating information (Faisal and Shaker 2017). Moreover, its application in generating CES studies remains limited. On the other hand, conducting in‐depth analysis and accurately quantifying the various factors driving changes in ESs are crucial for formulating effective regional management strategies (Luo et al. 2020). Land Use/Cover Change (LUCC) is considered a significant element and a primary driver of ecosystem change globally (Hu et al. 2024; Yang et al. 2021). Additionally, climate factors (Braun et al. 2019), topographic factors (Li et al. 2024a), and other elements have also been proven to be crucial in influencing ESs. Methods commonly employed for analyzing driving forces include regression models (Hu et al. 2023b; Sannigrahi et al. 2020), the Geodetector model (Guo et al. 2024; Li et al. 2025), machine learning (Shen et al. 2024), and scenario simulation (Huang et al. 2024), among others. The versatility of the Geodetector model across different domains makes the knowledge it generates applicable to multiple disciplines globally (Liang and Xu 2023). Meanwhile, the optimal parameter‐based geographical detector model (OPGD) (Song et al. 2020) significantly enhances the accuracy and effectiveness of spatial analysis, making it a promising method for identifying key impact factors of ESs. Furthermore, as the spatial scale changes, the key driving factors for ESs in the same region may also shift accordingly (Su et al. 2020). Therefore, selecting an appropriate scale is particularly crucial in research (Rong et al. 2022).

Exploring the patterns of ES changes and their driving mechanisms holds significant importance for CYP. Despite being the most economically developed region and the core of new urbanization development in Yunnan Province, its overall development level remains low. The regional GDP of the CYP urban agglomeration is ranked 17th among all 19 urban agglomerations in China, indicating a significant development gap. It is also among the regions most severely affected by drought‐stricken China (Qu et al. 2022), with pronounced human–land conflicts. CYP is currently at a critical stage of transformation, upgrading, and innovative development and must coordinate economic development and environmental protection to provide strong support for sustainable regional development. Therefore, there is a necessity to determine the demand for ESs with respect to the ecological characteristics of CYP, establish an operational and reliable CES assessment framework for this area, and identify the key drivers of ESs. The study results are expected to offer valuable references for eco‐environmental protection, natural resource management, and eco‐security assessment in CYP and similar regions.

## 2 Materials and Methods

### 2.1 Study Area

**FIGURE 1:**
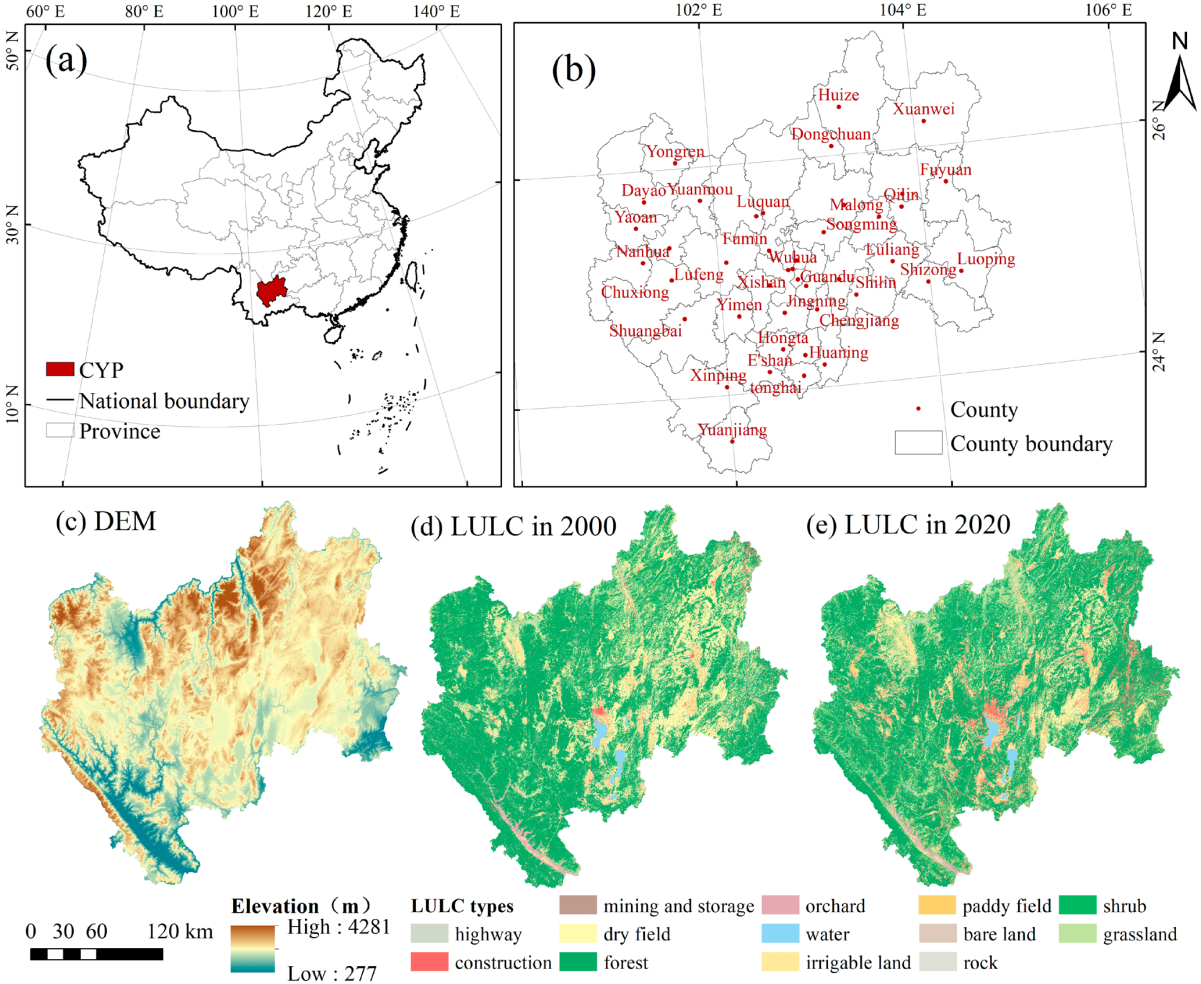
Maps of the study area (central Yunnan Province, CYP).

CYP, situated in the heart of Yunnan Province, possesses a unique geographic location (24.05°–25.46° N; 102.48°–103.04° E) and a rich endowment of natural resources. It covers an area of approximately 94,558km^2^ and has an average elevation ranging from 200 to 4000 m. The region is dominated by mountainous terrain, with mountains, rivers, dams, and lakes interspersed. CYP has vigorously developed modern agriculture, specialty cultural industries, and other sectors, contributing 53.70% of the province's GDP while occupying only 23.99% of land area (as of the end of 2021). It has emerged as one of the most dynamic and promising economic growth poles in Yunnan Province. CYP plays a vital role in the Western Development Program and serves as the core region for establishing a gateway to Southeast Asia. CYP holds a good development foundation and great development potential, and it has a substantial strategic position in the national development and opening‐up pattern (Figure 1).

### 2.2 Datasets and Data Processing

#### 2.2.1 Data Sources

This study used multiple datasets, including remote sensing images, LULC data, elevation data, etc. (Table 1), and all data were unified into WGS‐84 coordinates using ArcGIS.

**TABLE 1:** List of the basic data sources.

Category	Data types	Spatial resolution	Data source
Remote sensing data	Landsat5_TM Landsat7_ETM+ Landsat8_OLI	30 m	USGS (https://www.usgs.gov)
		30 m	
		30 m	
	MOD13Q1	250 m	NASA (https://earthdata.nasa.gov)
	Unconstrained individual countries 2000–2020 population UN adjusted count datasets	1 km	WorldPop hub (https://hub.worldpop.org)
	A Prolonged Artificial Nighttime‐light Dataset of China (1984–2020)	1 km	Derived from the Zhang et al. (2024a)
Meteorological data	1‐km monthly precipitation, temperature and potential evapotranspiration dataset for China from 1901 to 2021	1 km	National Earth System Science Data Center (http://www.geodata.cn/)
	1‐km annually Aridity index dataset for China from 1901 to 2021	1 km	
Soil data	Harmonized World Soil Database(HWSD)	30 arc‐second	Food and Agriculture Organization of the United Nations (https://www.fao.org/)
	Global Soil Organic Carbon Map	1 km	
Digital elevation data	Shuttle Radar Topography Mission (SRTM)	30 m	USGS (https://www.usgs.gov)

#### 2.2.2 LULC Data Processing

We established the classification system according to the actual situation of CYP and mainly used the Google Earth Engine (GEE) platform to produce the LULC classification data in CYP (Liu et al. 2022). (1) First, using the multitemporal Landsat5_TM, Landsat7_ETM+, and Landsat8_OLI satellite images, a cloud‐free or cloudless image was generated on the GEE platform as the original classification image. (2) Second, we performed visual interpretation based on Google satellite images and the original remote sensing data and used the semiautomatic selection principle to collect training sample data and partial validation sample data. (3) The remote sensing images were filtered, cloud masked, stitched, and cropped by date and fused with SRTM data to generate images with a higher accuracy. (4) To improve the classification accuracy, we incorporated auxiliary data into the classification process, such as NDVI, NDWI, and water vapor data. (5) Finally, we used the *Classifier.libsvm* classifier to supervise the classification of the image data and output the classification results. The accuracy was verified based on visual interpretation verification samples and field survey samples, and the overall accuracy reached 90%.

#### 2.2.3 Selection of Driving Factors

**FIGURE 2:**
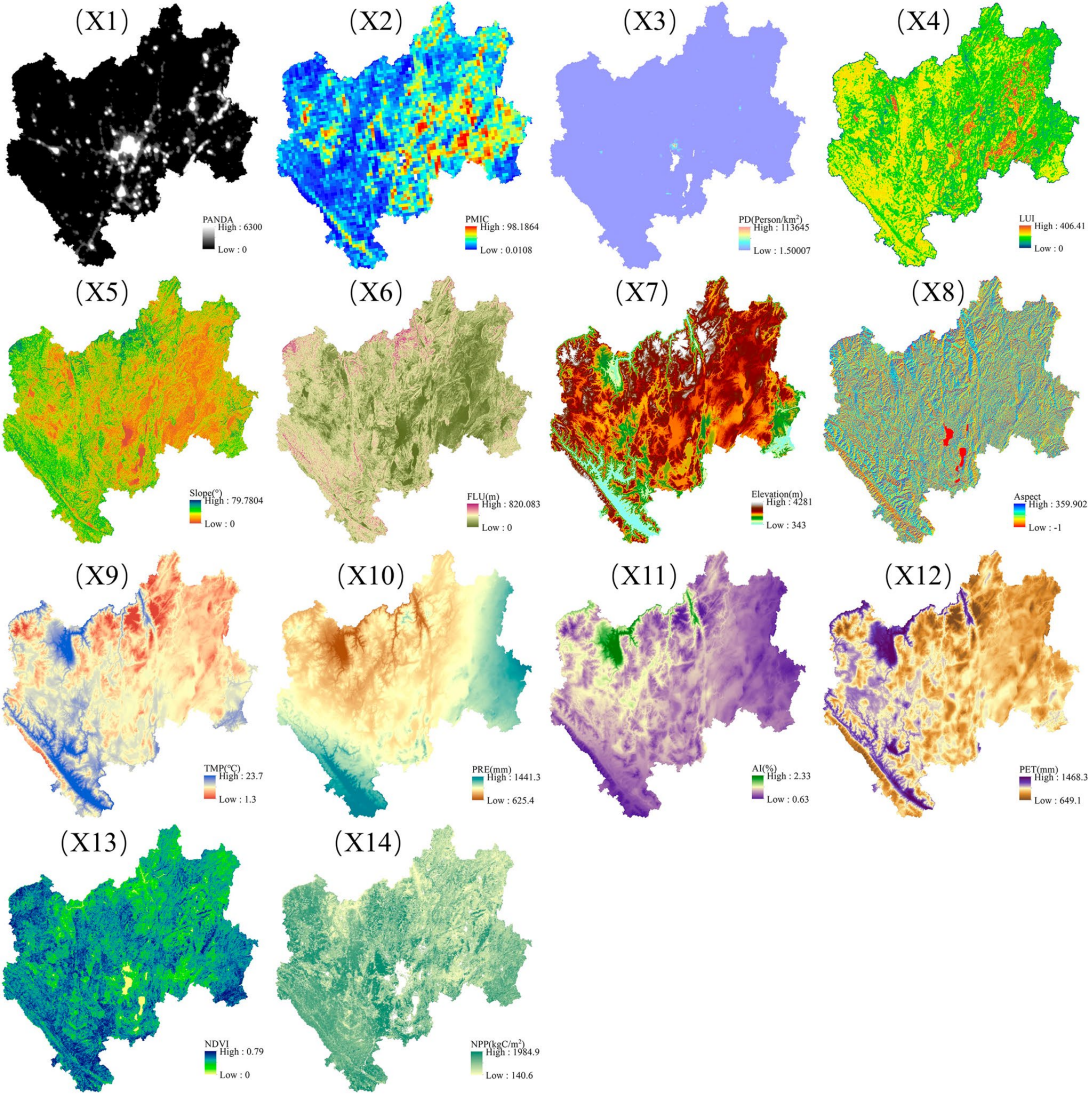
Spatial distribution of 14 drivers in CYP.

ES changes are complex and dynamic processes, and vegetation, climate change, topography, and human disturbances are all important driving factors. We selected 14 key influence factors (Figure 2) as driving factors based on their availability and relevance to our research, including precipitation, temperature, NDVI, and others (Table S1).

### 2.3 Methods

**FIGURE 3:**
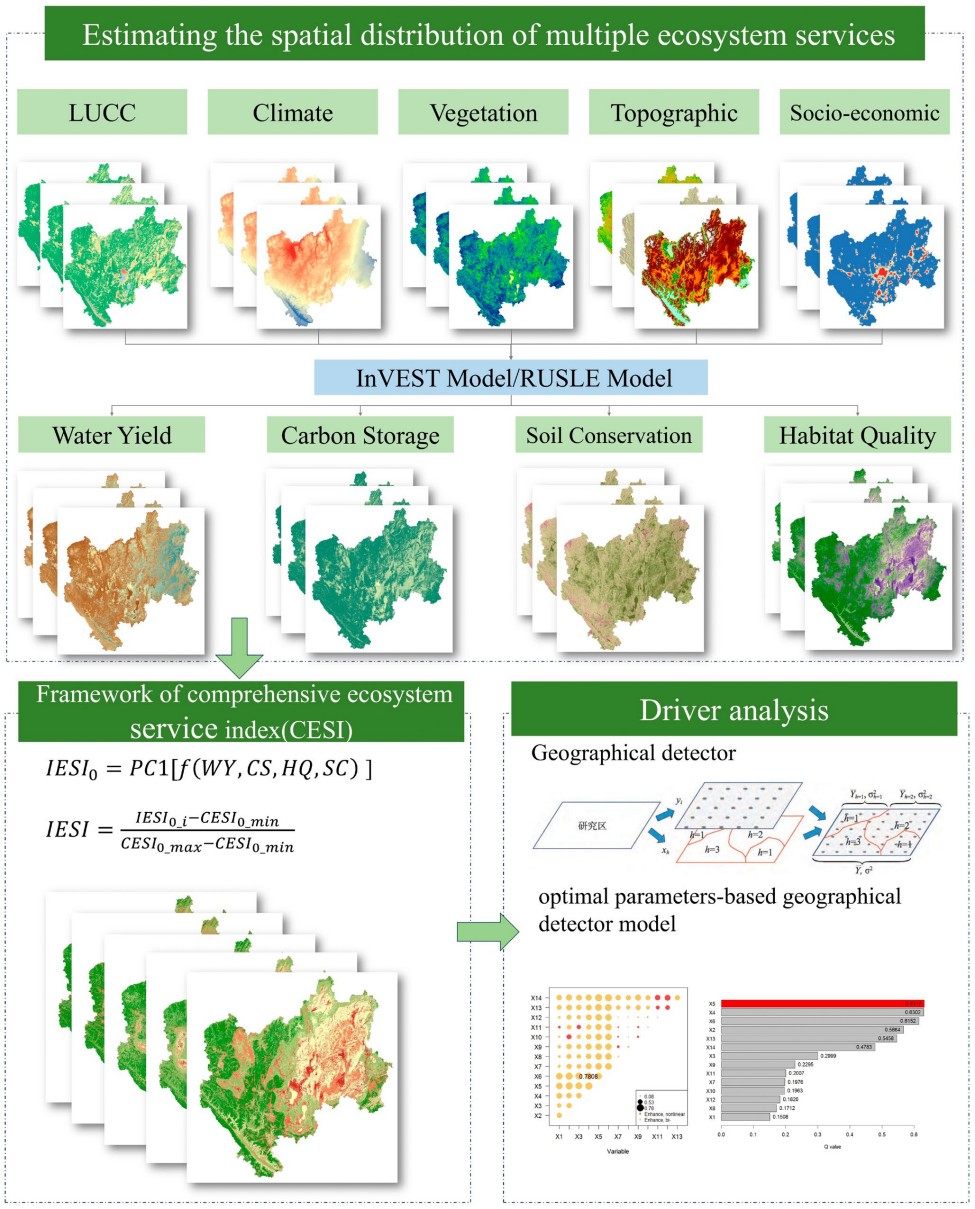
Research flow chart.

This study focuses on three main aspects. First, we selected WY, SC, CS, and HQ as key indicators related to human well‐being to assess ESs using the InVEST and RUSLE models from 2000 to 2020 under the contexts of water scarcity, severe soil erosion, and habitat degradation in CYP. Second, we proposed an integrated method based on PCA to construct IESI. Finally, we applied the OPGD model to identify the main driving factors in CYP. The organizational flow chart is shown in Figure 3.

#### 2.3.1 Methods for Assessing the Amount of Ess

This study quantified these ESs through simulations using the InVEST and RUSLE models. The appendix provides detailed parameters and the rationale of the model.

##### 2.3.1.1 Water Yield

The WY assessment module is based on Budyko's (1974) coupled water and energy balance assumptions. It uses the following equation:

(1) $$Y\left(x\right)=\left(1-\frac{\mathrm{AET}\left(x\right)}{P\left(x\right)}\right)P\left(x\right)$$

where $Y\left(x\right)$, $\mathrm{AET}\left(x\right)$, and $P\left(x\right)$ are the annual WY, actual evapotranspiration, and precipitation of grid x (mm) in CYP, respectively.

##### 2.3.1.2 Carbon Storage

We used the Carbon Storage and Sequestration module, which serves as a library‐based method (Sharp et al. 2018) that calculates total regional carbon storage to calculate the CS in CYP. The calculation equation is as follows:

(2) $$C_{\mathrm{tot}}=C_{\text{above}}+C_{\text{below}}+C_{\text{soil}}+C_{\text{dead}}$$

where $C_{\mathrm{tot}}$ is the total regional CS; $C_{\text{above}}$ is the aboveground CS; $C_{\text{below}}$ is the belowground root CS; $C_{\text{soil}}$ is the soil CS; and $C_{\text{dead}}$ is the dead organic matter CS.

##### 2.3.1.3 Habitat Quality

The Habitat Quality module obtains habitat distribution characteristics by linking various LULC types to sources of environmental threats (Sharp et al. 2018) and is calculated as follows:

(3) $$Q_{\mathrm{xj}}=H_{j}\left(1-\left(\frac{D_{\mathrm{xj}}^{z}}{D_{\mathrm{xj}}^{z}+K^{z}}\right)\right)$$

(4) $$D_{\mathrm{xj}}=\underset{r=1}{\sum^{R}}\underset{y=1}{\sum^{Y_{r}}}\left(\frac{W_{r}}{\sum_{r=1}^{R}W_{r}}\right)r_{y}i_{\mathrm{rxy}}\beta_{x}S_{\mathrm{jr}}$$

where $Q_{\mathrm{xj}}$ is the HQ of raster x in LULC type j; $D_{\mathrm{xj}}$ is the total threat level of raster x in LULC type j; K and Z are scaling factors; $H_{j}$ is the habitat suitability of LULC type j; R denotes the stress factor; $Y_{r}$ is the number of grids occupied by stressor r; $W_{r}$ indicates the weight of the stressor, with values from 0 to 1; $r_{y}$ is the stressor value of grid y; $i_{\mathrm{rxy}}$ denotes the stress level of stress factor value $r_{y}$ of raster y to habitat raster x; $\beta_{x}$ denotes the accessibility level of raster x; and $S_{\mathrm{jr}}$ denotes the sensitivity of habitat type j to stress factor r.

##### 2.3.1.4 Soil Conservation

The Revised Universal Soil Loss Equation (RUSLE) (Fang et al. 2015) was used to estimate the SC in CYP. The calculation equation is as follows:

(5) $$A=R\times K\times \mathrm{LS}\times \left(1-C\times P\right)$$

where A is the SC amount per unit area (t/(hm^2^‐a)); R is the rainfall erosion force factor (MJ·mm/(hm^2^·h·a)); K is the soil erodibility factor (t·hm^2^·h/(MJ·hm^2^·mm)); LS is the slope gradient and slope length factor; C is the vegetation cover and management factor; and P is the soil and water conservation measure factor.

#### 2.3.2 Integrated Ecosystem Service Index (IESI) Construction

The IESI for CYP was calculated using the PCA (Bro and Smilde 2014) method. The PCA objectively determines the weight values, enabling the conversion of four individual indicators (WY, CS, HQ, and SC) into a composite index, which provides an objective evaluation of ES in CYP from 2000 to 2020.

First, the four ESs are transformed into dimensionless standard values:

(6) $${\mathrm{NI}}_{i}=\frac{{\text{Indicator}}_{i}-{\text{Indicator}}_{\min}}{{\text{Indicator}}_{\max}-{\text{Indicator}}_{\min}}$$

where ${\mathrm{NI}}_{i}$ is the standardized value of an indicator; ${\text{Indicator}}_{i}$ is the value of the indicator in pixel i; ${\text{Indicator}}_{\max}$ is the maximum value and ${\text{Indicator}}_{\min}$ is the minimum value.

Secondly, the first principal component (PC1) is used to construct the ${\text{IESI}}_{0}$, and the weights of each indicator are objectively determined by their contribution to PC1.

(7) $${\text{IESI}}_{0}=\mathrm{PC}1\left[f\left(\mathrm{WY},\mathrm{CS},\mathrm{HQ},\mathrm{SC}\right)\right]$$

Finally, the ${\text{IESI}}_{0}$ can also be standardized:

(8) $$\text{IESI}=\frac{{\text{IESI}}_{0\_i}-{\text{IESI}}_{0\_\min}}{{\text{IESI}}_{0\_\max}-{\text{IESI}}_{0\_\min}}$$

IESI is the constructed index with values ranging between 0 and 1. The higher the IESI value, the greater the overall ES capacity.

#### 2.3.3 OPGD Model

##### 2.3.3.1 Parameter Optimization

Parameter optimization includes spatial scale and spatial discretization (Song et al. 2020). Based on the scope of CYP and considering relevant research (He et al. 2024; Liu et al. 2023b), the sample area should be between two and five times the average patch area (Xie et al. 2017). Therefore, we constructed a total of nine scales from 1000 m to 5000 m at 500‐m intervals. Second, the *q*‐value of each driving factor under different classification methods and numbers of classifications was calculated by the OPGD model. Next, the combination with the highest *q*‐value was selected for optimal spatial discretization. Finally, the nine different spatial scales were compared, and the grid with consistently high *q*‐values across factors and stable factor *q*‐value rankings was selected as the optimal geographical detection scale.

##### 2.3.3.2 Geodetector (GD) Model

The GD model is a statistical analysis method that detects spatial divergence and quantitatively explains driving forces (Wang and Xu 2017).

1. Divergence and factor detector. It is primarily used to detect the spatial divergence of Y and to detect the extent to which factor X explains the spatial divergence of Y. The expression is

(9) $$q=1-\frac{\underset{h=1}{\sum^{L}}N_{h}\sigma_{h}^{2}}{N\sigma^{2}}$$

In Equation (9), take the driving factor X1 as an example: q indicates the explanatory power of X1 of CES spatial divergence with the threshold value of [0,1]; h indicates the sub‐region of X1; $N_{h}$ and N represent the number of units in the sub‐region of X1 and in the whole region; the variance of the sub‐region of X1 and the whole region are expressed as σ and σh.

2 Interaction detector. It is used to detect interaction relationships between various factors and to determine whether the interaction between independent variables amplifies or diminishes their effect on the dependent variable. Five intervals of factor interaction are identified (Table 2):

**TABLE 2:** Interaction between explanatory variables.

Description	Interaction
$q\left(X1\cap X2\right)<\min\left[q\left(X1\right),q\left(X2\right)\right]$	Weaken, nonlinear
$\min\left[q\left(X1\right),q\left(X2\right)\right]<q\left(X1\cap X2\right)<\max\left[q\left(X1\right),q\left(X2\right)\right]$	Weaken, uni—
$q\left(X1\cap X2\right)>\max\left[q\left(X1\right),q\left(X2\right)\right]$	Enhance, bi—
$q\left(X1\cap X2\right)=q\left(X1\right)+q\left(X2\right)$	Independent
$q\left(X1\cap X2\right)>q\left(X1\right)+q\left(X2\right)$	Enhance, nonlinear

## 3 Results and Analysis

### 3.1 Characteristics of Spatio‐Temporal Changes in Single ES

**FIGURE 4:**
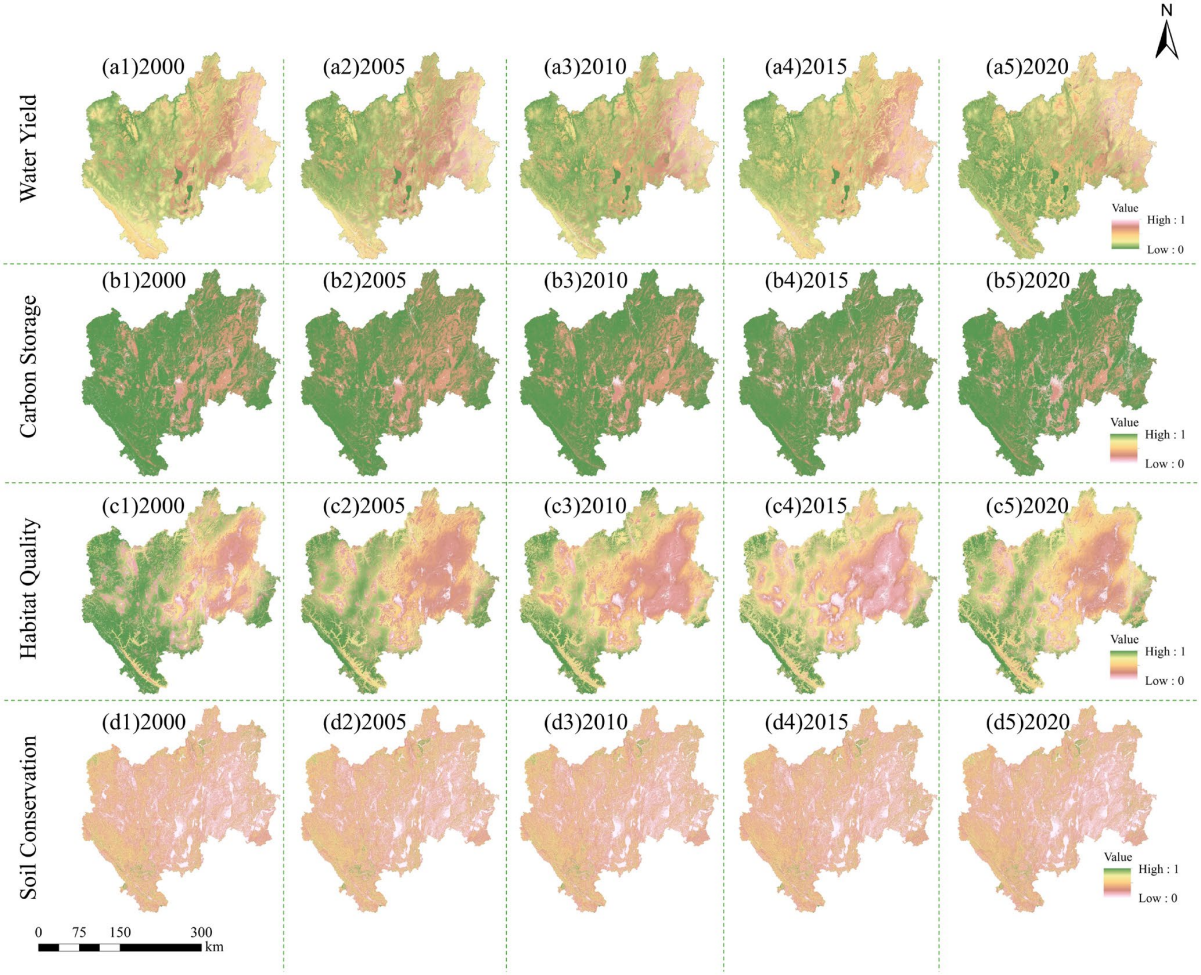
Spatio‐temporal distribution of WY, CS, HQ, and SC in CYP.

ESs in CYP exhibited significant regional variability, minimal inter‐annual changes, and high spatial distribution stability from 2000 to 2020 (Figure 4). Areas with high WY services were mainly concentrated in central Qujing, including Fuyuan County, Xuanwei City, and Zhanyi County, while the low WY areas were located in Shuangbai, Yimen, and Lufeng in Chuxiong Prefecture, as well as in the water bodies of Dianchi Lake and Fuxian Lake. This distribution exhibited a spatial pattern with higher values in the east and lower values in the west. The average WY index decreased from 0.2525 in 2000 to 0.2394 in 2020. The spatial distribution of CS exhibited a pattern opposite to that of WY. Areas with high CS were concentrated in mountainous regions with complex terrain, such as the Wumeng Mountain and Ailao Mountain in western Chuxiong and within Yuxi, where evergreen broad‐leaved forests and alpine meadows are prevalent. In contrast, low CS areas are primarily located in water bodies, cities, and agricultural plantations. The average CS showed a fluctuating inter‐annual trend, with values of 0.9078 in 2000, 0.8943 in 2005, 0.9053 in 2010, 0.8948 in 2015, and 0.9133 in 2020. HQ and CS shared a similar spatial distribution, with high‐value areas located in regions with abundant forest resources and natural water bodies such as Dianchi Lake and Fuxian Lake. Conversely, low‐value areas were concentratedly distributed along rivers and lakes, particularly in regions of socioeconomic development, such as Qujing and Kunming, where the dominant LULC types are farmland and construction land. From 2000 to 2015, the average HQ steadily declined from 0.6640 to 0.5496, but increased to 0.5849 between 2015 and 2020. During the study period, the average SC increased from 0.0405 in 2000 to 0.0489 in 2005, but subsequently decreased to 0.0360 by 2020. Most areas in CYP had relatively low SC values, with minimal changes in spatial distribution. Low SC areas were primarily located in the plateau lake basin regions and the Jinsha River's hot‐dry valley, including southern Kunming, central Qujing, and northern Yuxi. These regions are characterized by high population density, higher levels of socioeconomic development, but low vegetation cover. High SC areas were concentrated in northern and southwestern Chuxiong, northern Yuxi, and northern Qujing, where the predominant LULC are forest and grassland.

### 3.2 Characteristics of IESI Changes

#### 3.2.1 Spatial Pattern of IESI

**FIGURE 5:**
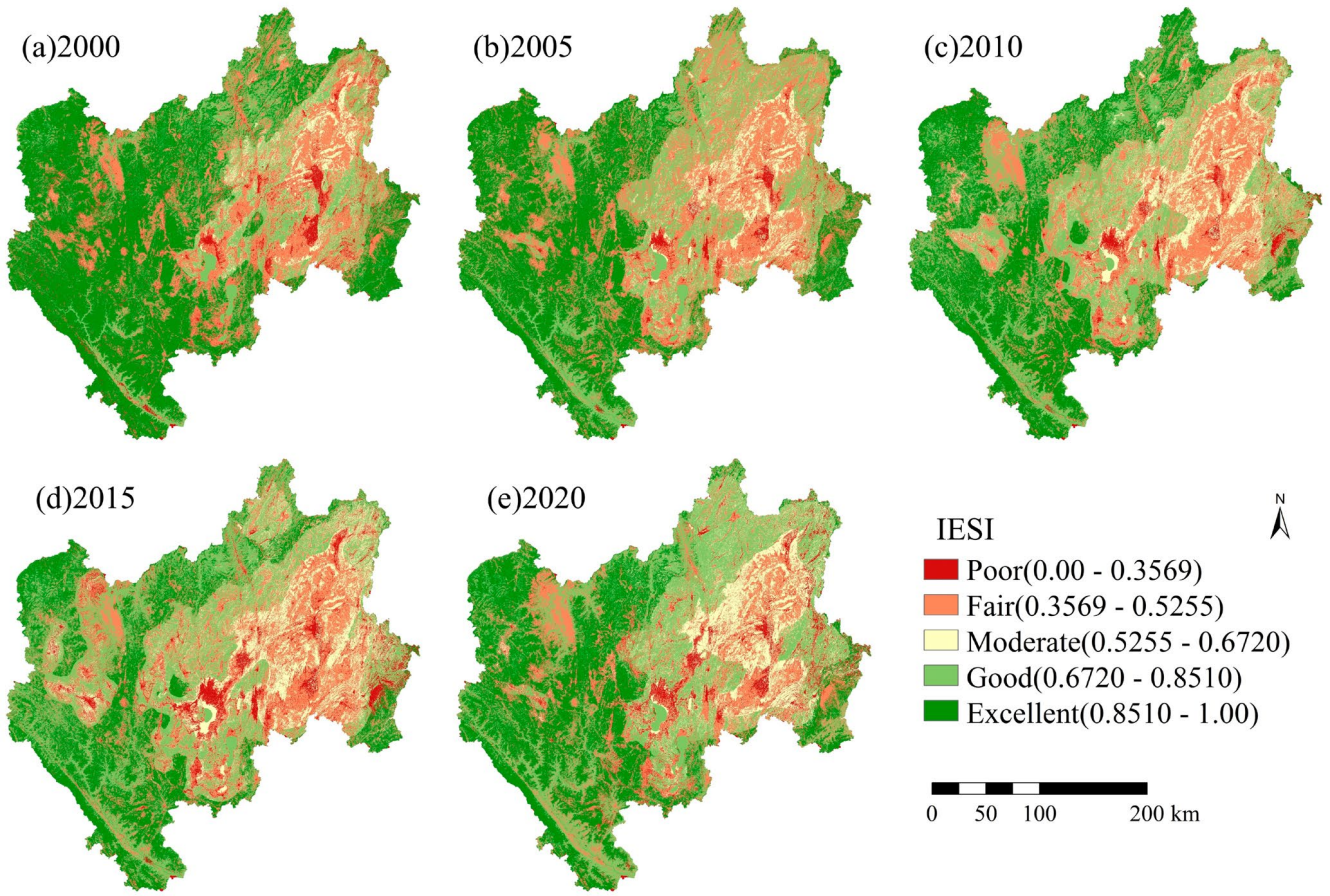
Spatial distribution of IESI in the CYP.

We applied PCA to analyze the four ES indicators (Table 3). The results revealed that PC1 had the largest eigenvalue, with a contribution rate exceeding 79% throughout the study period, which can effectively integrate the information from the four indicators. Therefore, PC1 was used to construct the IESI in this study. From 2000 to 2020, the average IESI ranged between 0.6412 and 0.7160, indicating an overall decline in CES. Specifically, the IESI decreased steadily from 0.7338 in 2000 to 0.6650 in 2015, followed by an increase to 0.6992 in 2020, suggesting that CES in the CYP initially declined and then gradually improved. Spatially, the IESI in CYP exhibited a pattern with greater values in the northwest and diminished values in the southeast (Figure 5). Specifically, areas with poor and fair IESI levels were primarily concentrated in eastern Kunming and central Qujing, regions characterized by higher levels of socioeconomic development and abundant available land resources. In addition, the Jinsha River's hot‐dry valley in Chuxiong Prefecture also displayed relatively poor CES capacity. Conversely, areas with good and excellent IESI levels were predominantly situated in the southwestern part of CYP, particularly within the Ailao Mountain range, which had a high level of vegetation cover and rich biodiversity.

**TABLE 3:** Principal component analysis of four factors.

Year	Index	PC1	PC2	PC3	PC4
2000	WY	−0.3942	0.9178	−0.0413	−0.0249
	CS	0.3306	0.1854	0.9227	0.0703
	HQ	0.8575	0.3500	−0.3737	−0.0507
	SC	0.0105	0.0277	−0.0852	0.9959
	Eigenvalues	0.0731	0.0060	0.0024	0.0007
	Percent of Eigenvalues	88.9114	7.3181	2.9718	0.7987
2005	WY	−0.4617	0.2362	0.8547	−0.0252
	CS	0.3464	−0.0699	0.9345	0.0423
	HQ	0.8165	0.5119	−0.2619	−0.0523
	SC	0.0165	0.0514	−0.0474	0.9974
	Eigenvalues	0.0661	0.0063	0.0027	0.0008
	Percent of Eigenvalues	87.0402	8.2952	3.5552	1.1094
2010	WY	−0.4916	0.8273	0.2714	−0.0151
	CS	0.3288	−0.1116	0.9373	0.0293
	HQ	0.8061	0.5489	−0.2159	−0.0476
	SC	0.0213	0.0419	−0.0337	0.9983
	Eigenvalues	0.0606	0.0079	0.0036	0.0005
	Percent of Eigenvalues	83.4760	10.8427	4.9682	0.7131
2015	WY	−0.4593	0.8801	0.1200	−0.0099
	CS	0.3437	0.0520	0.9372	0.0270
	HQ	0.8189	0.4714	−0.3254	−0.0371
	SC	0.0166	0.0249	−0.0362	0.9989
	Eigenvalues	0.0618	0.0098	0.0038	0.0005
	Percent of Eigenvalues	81.3599	12.8989	5.0593	0.6819
2020	WY	−0.4635	0.8853	0.0382	−0.0086
	CS	0.3262	0.1307	0.9355	0.0363
	HQ	0.8238	0.4460	−0.3481	−0.0356
	SC	0.0136	0.01874	−0.0461	0.9987
	Eigenvalues	0.0622	0.0081	0.0037	0.0006
	Percent of Eigenvalues	83.3806	10.8389	5.0171	0.7634

#### 3.2.2 Characteristics of Temporal Changes in IESI

**FIGURE 6:**
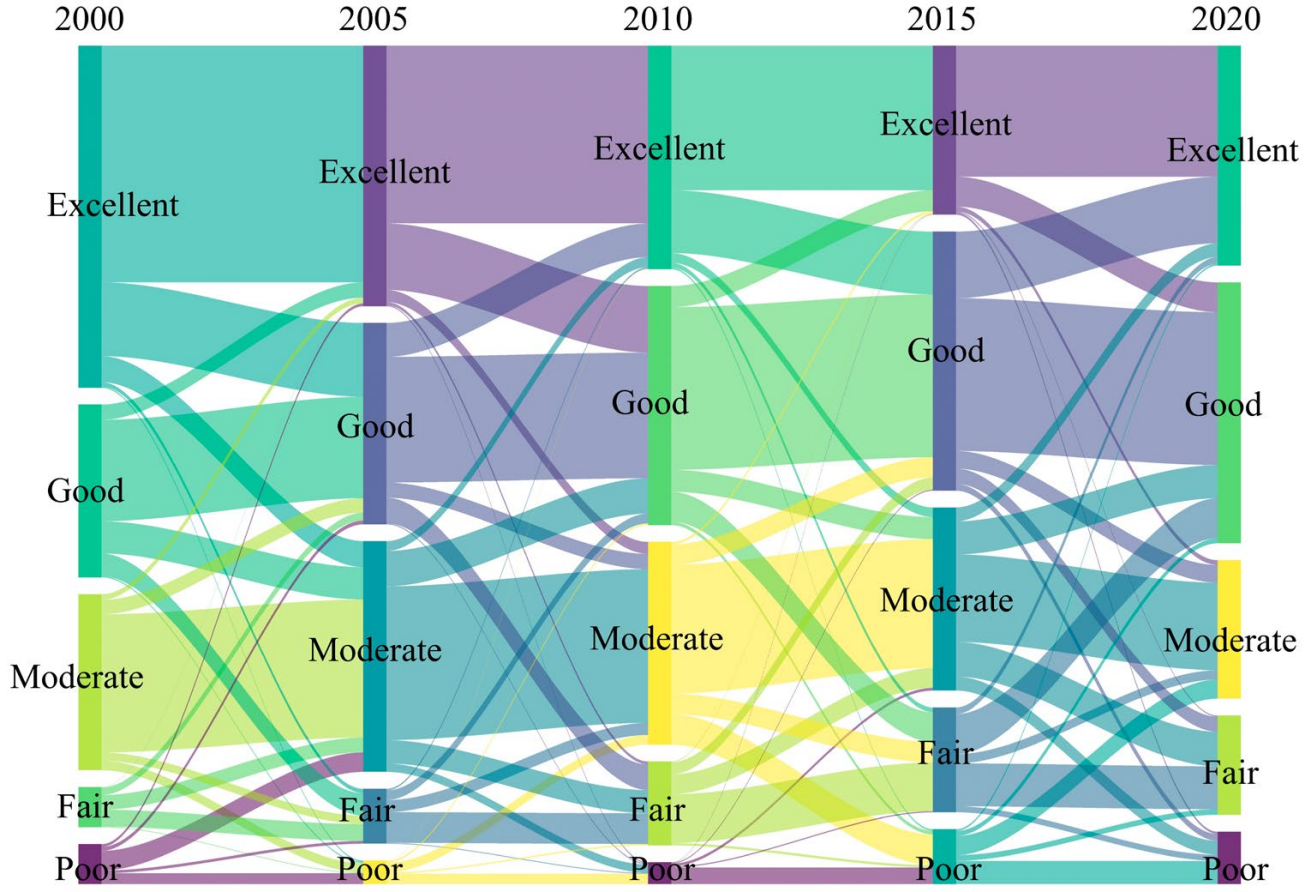
Sankey diagram of CES level transfer in the CYP during the study period.

The transition map of IESI levels in CYP from 2000 to 2020 is shown in Figure 6. Throughout the study period, the IESI was primarily at excellent, good, and moderate levels, with significant area shifts occurring between these three categories. Specifically, from 2000 to 2005, there was an overall decline in IESI levels, marked by a reduction in the areas classified as excellent and poor, and an increase in areas classified as good, moderate, and fair. From 2005 to 2010, the overall IESI level continued to decline, with the proportion of area at the excellent level dropping to 28.99%, mainly shifting to the good level, which became the largest category at 30.99%. From 2010 to 2015, the area at the good level continued to increase, reaching 33.58%, while the area at the excellent level decreased to 21.93%. From 2015 to 2020, the IESI level improved, with decreases in the areas classified as poor and fair, and an increase in the area classified as excellent to 28.60%. Overall, from 2000 to 2015, the area of IESI classified as excellent consistently decreased, while the areas classified as moderate and good increased. During the 2015–2020 period, the IESI showed an overall improvement trend.

### 3.3 IESI Driver Analysis

#### 3.3.1 Optimal Grid Scale

**FIGURE 7:**
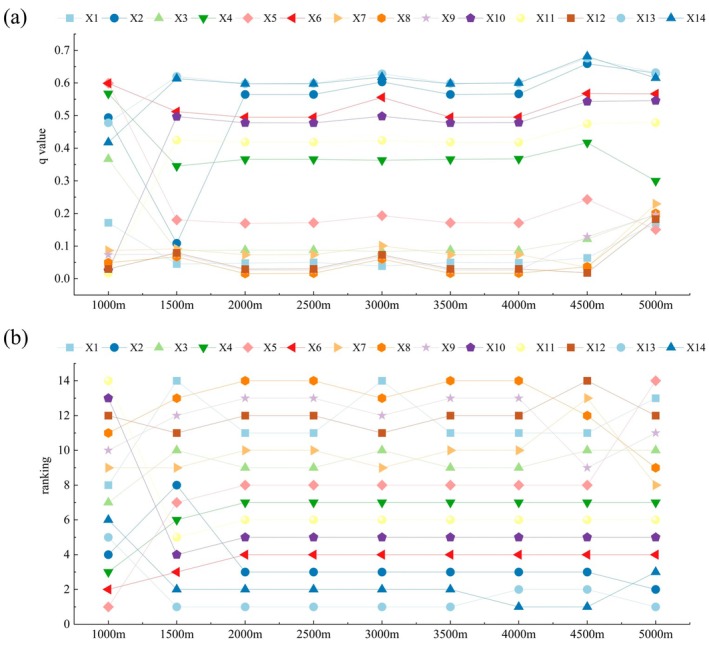
Comparison of factor detection results at different scale grids.

There is significant variation in the influence of different factors on IESI at different grid scales. We selected 9 sets of scale grids with intervals of 500 m, 1000 m to 5000 m for sampling and analyzing IESI and driving factors, and used the OPGD model to compare the differences in the modeling results at different grid scales. Finally, the 4500 m × 4500 m grid with high factor *q*‐values and stable *q*‐value ordering of the factors was selected as the optimal scale (Figure 7).

#### 3.3.2 Factor Detection

**FIGURE 8:**
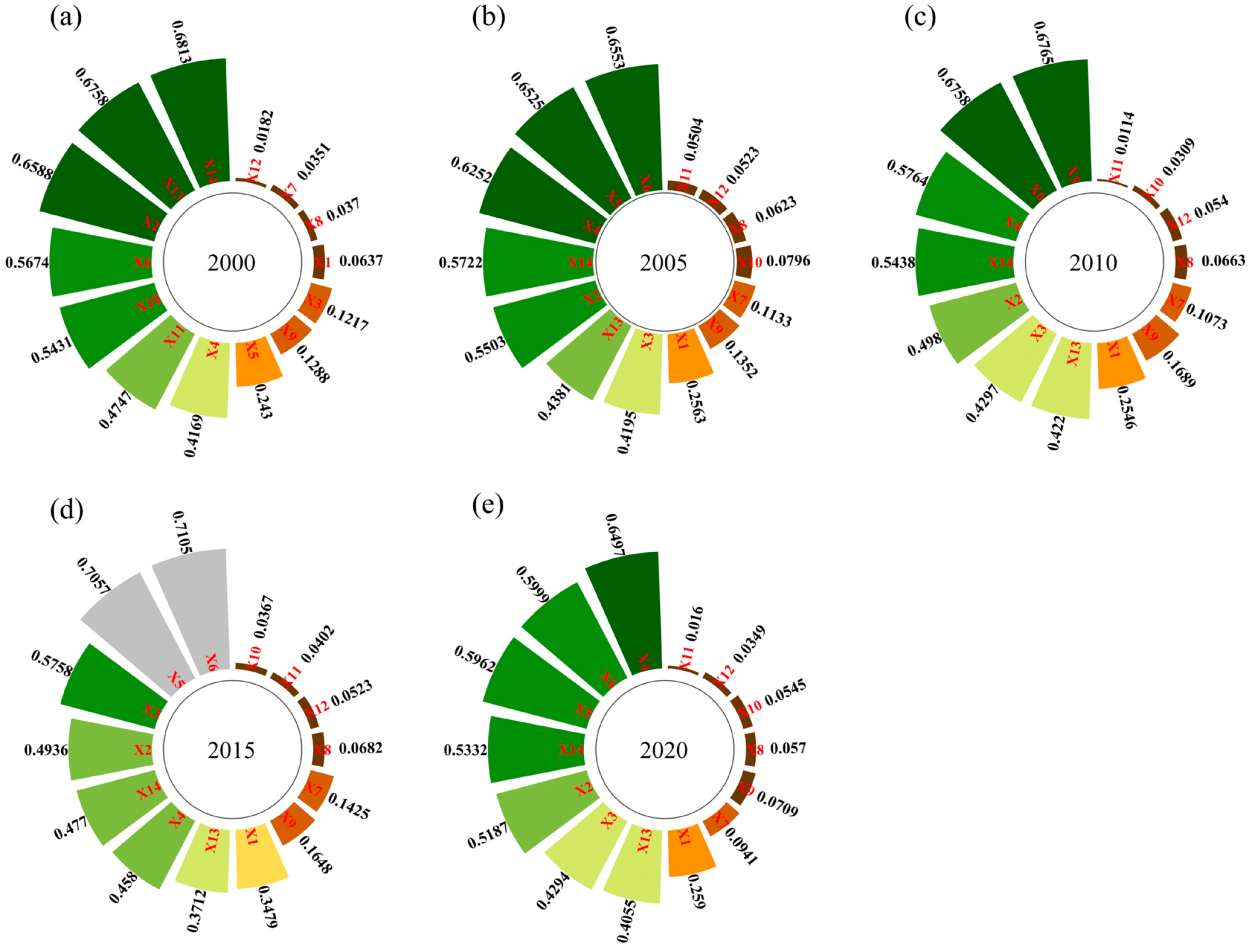
Factor detection results of IESI spatial divergence in CYP from 2000 to 2020.

The q‐values of the driving factors were statistically analyzed, and all passed the significance test at the 0.01 level, indicating a significant influence on the spatial divergence of IESI (Figure 8). Overall, RDLS (X6) contributed the most to the spatial divergence of IESI, with an average *q*‐value of 0.6418, with slope (X5) and NDVI (X14) ranking next, having *q*‐values of 0.5748 and 0.5615, respectively. Evaporation (X12) contributed the least (*q* = 0.0423), while aspect (X8) and elevation (X7) also had relatively minor contributions, with *q*‐values of 0.0581 and 0.0984, respectively. Additionally, the contributions of the driving factors varied considerably across different years. In 2000, NDVI had the highest contribution (*q* = 0.6813), followed by NPP (X13) (*q* = 0.6758) and the land reclamation rate(X2) (*q* = 0.6588). However, in 2005, 2010, and 2020, the RDLS had the highest contribution, followed by slope, land use intensity (LUI), and NDVI, with q‐values greater than 0.50, indicating that these factors had a greater influence than others. In 2015, population density (*q* = 0.4936) surpassed LUI (*q* = 0.4580) as the third most influential factor. The effects of the aridity index, evaporation, precipitation, and aspect were all relatively minor during the study period.

#### 3.3.3 Interaction Detection

**FIGURE 9:**
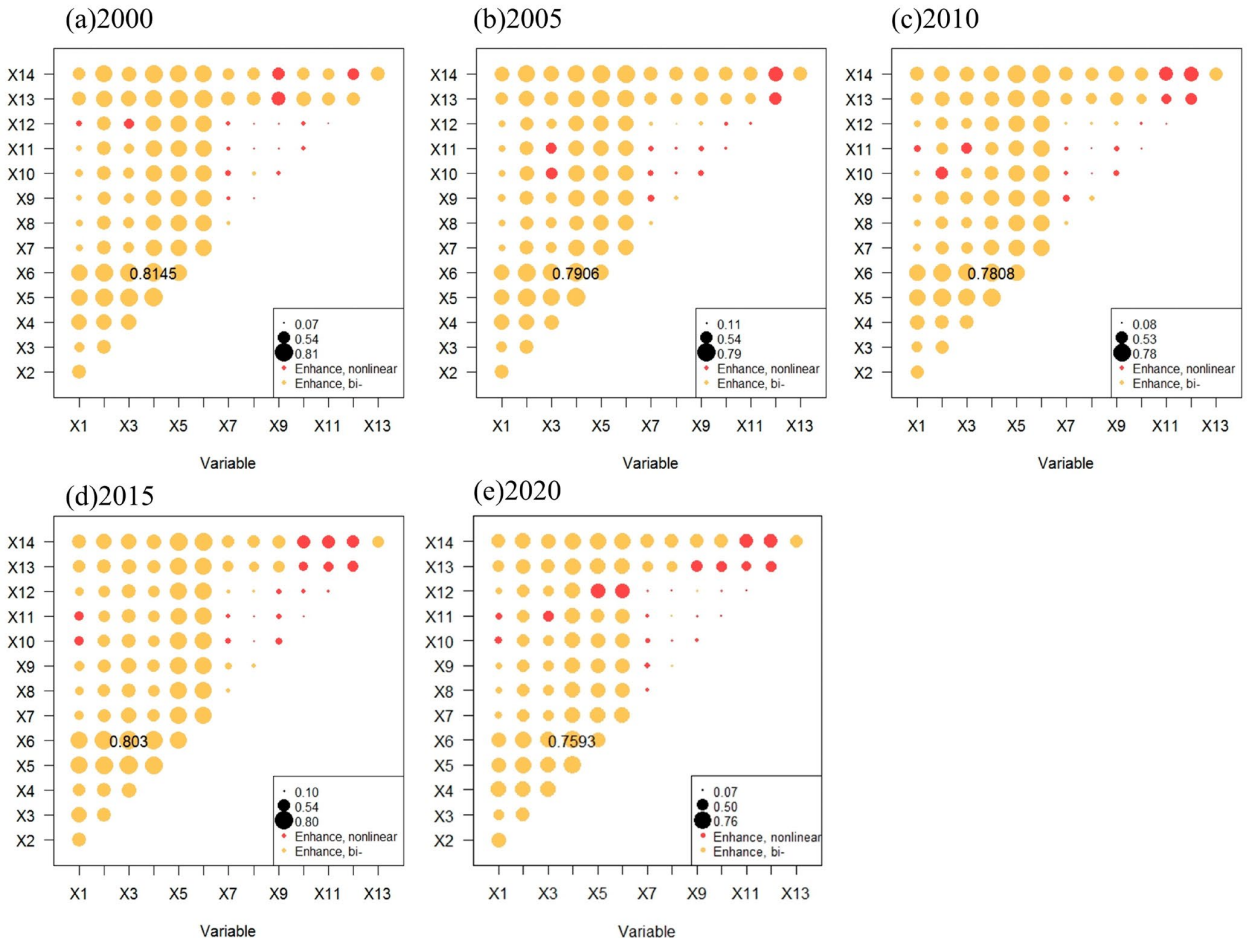
Interaction detection results of any two influence factors on IESI changes.

The *q*‐values for the interaction between any two influencing factors on the spatial divergence of IESI in CYP were all greater than those of the individual factors from 2000 to 2020. As shown in Figure 9, approximately 1/5 of the factors exhibited a nonlinear enhanced interaction, while the remainder showed bivariate enhancement, with no weakening or independent effects observed. In 2015, the highest interaction *q*‐value occurred between population density (X3) and the RDLS, reaching 0.8030. In other years, the highest interaction q‐values were consistently between LUI (X4) and the RDLS, with values of 0.8145, 0.7906, 0.7808, and 0.7593, respectively. These findings suggest that the spatial variation of IESI in CYP is significantly influenced by the interactions between RDLS, LUI, and population density, rather than by any single factor alone.

## 4 Discussion

### 4.1 Advantages and Applicability of Constructing the IESI

**FIGURE 10:**
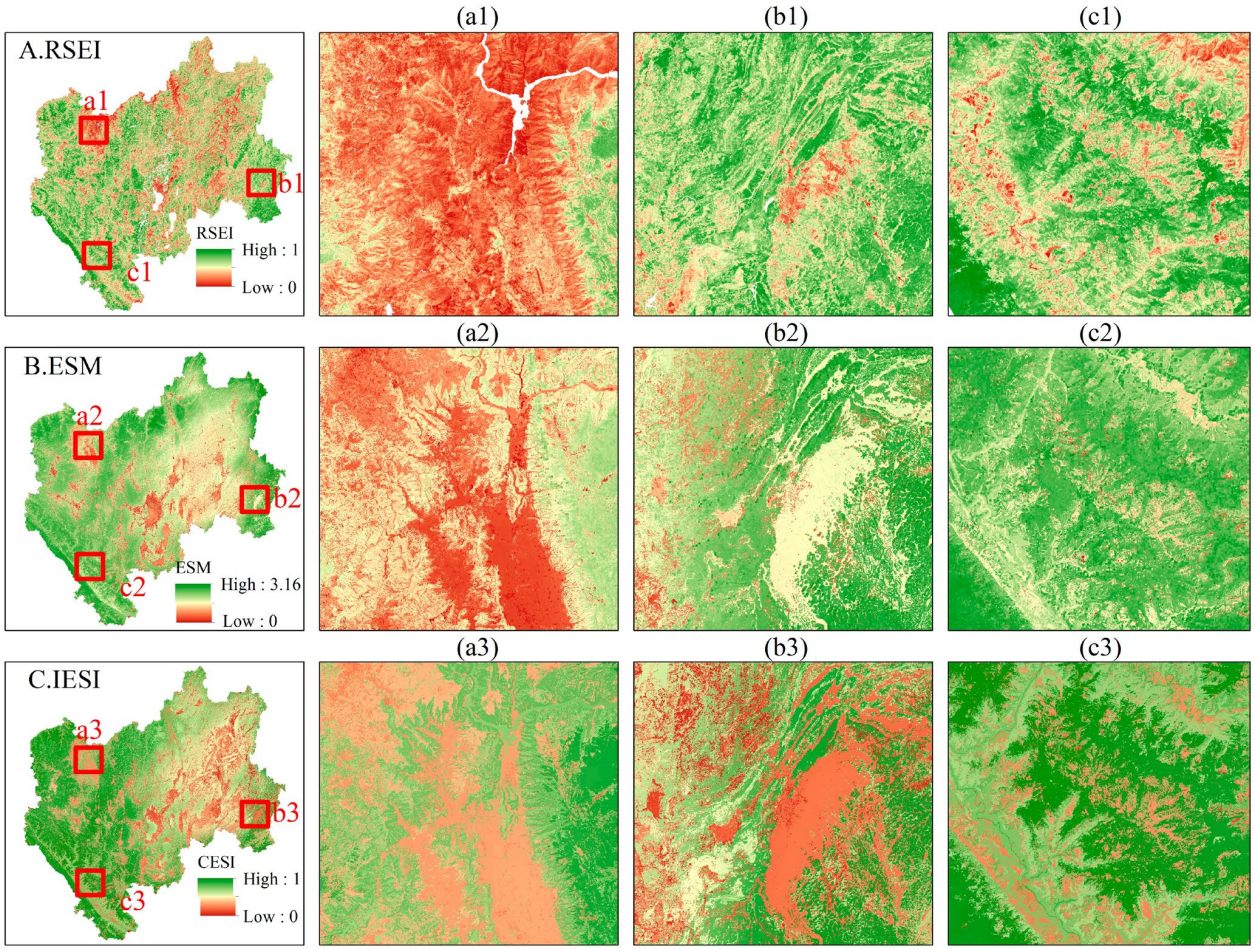
Comparison of IESI with RSEI and ESM results.

The rationality and scientificity of the model can be verified through the results of the CES assessment. Given the complexity and spatial heterogeneity of regional ecosystems, directly determining the reliability of the evaluation results is challenging (Li et al. 2023). Therefore, this study introduces the Remote Sensing Ecological Index (RSEI) (Xu 2013) and the ecosystem service multifunctionality (ESM) index (Liu et al. 2021) for comparative validation of the results. Rapid assessment of ecological quality using RSEI is reliable, and it is widely used in related fields (Xu 2013). Meanwhile, the ESM is one of the main approaches for quantitatively evaluating the capacity of ESs. Taking 2020 as an example, we selected three areas for comparative analysis: (a) the dry‐hot valley of Yuanmou, (b) the Luoping Basin, and (c) the mountainous and river valley areas of Xinping County. The spatial distribution of IESI evaluation results was generally consistent with the RSEI (Figure 10), effectively reflecting the CES in CYP. Furthermore, previous studies have demonstrated that RSEI is not suitable for large water bodies (Xu 2013), despite the crucial role that watersheds play in maintaining ES functions (Zheng et al. 2024). Additionally, ESM tends to underestimate the ES provided by water bodies to some extent. In summary, we conclude that using PCA to construct IESI enables the objective weighting and scientific integration of various ESs, which effectively reflects the ecosystem's capacity to provide multiple services. Furthermore, the ISEI is a relative value characterized by dynamism. It not only can quantify the CES in the CYP but can also function as a key indicator for evaluating the CES in other regions. Consequently, it can play a fundamental role in facilitating the coordinated development of society and the ecosystem.

### 4.2 Spatio‐Temporal Changes of CES in CYP

The average IESI value in CYP over the past 20 years was 0.6982, and IESI values were higher in the western mountainous areas, which include the Ailao Mountains and Wuliang Mountains. These regions are key ecological function zones in Yunnan Province, known for their significant ES functions (Qi et al. 2012). In contrast, lower IESI values were found in the dry‐hot valleys dominated by the Jinsha River and the karst regions in CYP, which is consistent with previous findings (Chen et al. 2022; Ran et al. 2021). During the study period, regions with improved IESI were chiefly centered in the eastern areas, where the most significant improvements were observed in eastern Xuanwei City and Fuyuan County. This reflects that the effectiveness of key ecological projects has been fully demonstrated, resulting in an overall enhancement of the ecological environment in the karst areas (He et al. 2019). At the same time, central Kunming, northern Qujing, and southern Yuxi have shown a declining trend in IESI. These areas have high vegetation coverage and diverse vegetation types, but unsustainable development activities have led to severe degradation of secondary natural forests (Li et al. 2024a). Although the area of plantations has increased (Cheng et al. 2024), this increase has not been sufficient to offset the overall decline, which has resulted from the loss of natural forests. Additionally, the IESI decreased by 0.0688 overall, indicating a general decline in CES. The analysis reveals that the trend of CES in CYP can be divided into two phases: “decreasing” and “increasing,” with 2015 marking a turning point. Before 2015, long‐term, large‐scale development and insufficient environmental protection measures (Peng et al. 2018) contributed to a widespread reduction in regional ES (Ran et al. 2021). From 2000 to 2015, CYP experienced accelerated planning and rapid urbanization, resulting in growing tensions between economic growth and resource scarcity, as well as between rapid urban expansion and increased environmental pressure. Furthermore, from 2001 to 2010, CYP was a key area affected by drought events in Southwest China (Ge et al. 2023). In 2014, Yunnan Province developed and implemented the “Yunnan Province Main Functional Area Planning,” which subsequently led to some improvement in CES in CYP. Therefore, both human and natural factors have contributed to the changing trends in CES.

### 4.3 Differences in the Impact of Factors

The spatio‐temporal fluctuations in CES result from the interplay of natural and human factors (Nelson et al. 2006). Therefore, we used the OPGD model to identify the driving factors behind IESI. In the study, the *q*‐values of the factor detector varied with scale, demonstrating a clear scale effect (Liu et al. 2023b). Based on the results from Section 3.1, the 4500 m × 4500 m spatial grid is the optimal spatial unit for assessing the impact of human activities and changes in the natural environment on the CES capacity in CYP. Overall, vegetation factors had the greatest combined impact on IESI, human activities and topographical factors contributing less. Specifically, the RDLS (*q* = 0.6418) is the variable most strongly correlated with IESI changes, succeeded by slope (*q* = 0.5748) and NDVI (*q* = 0.5615). This is because topography significantly influences regional ecological processes and pattern changes, making it one of the pivotal drivers of CES spatial divergence (Li et al. 2024a). In CYP, small undulating subalpine and mountains occupy a large area, and the overall geomorphic form is relatively fragmented (Liu and Wang 2017). The steep mountainous topography is a key factor affecting soil erosion in the study area (Li et al. 2024a). Additionally, vegetation is also a crucial factor affecting ESs, and increasing vegetation cover is a commonly used approach to improve and restore ESs (Ma et al. 2021). Ecological management projects such as returning farmland to forest and grassland and building shelterbelts are vigorously promoted in the study area, playing a key role in the improvement of CS, SC, and WY (Li et al. 2024a). Although climate change is acknowledged as a major driver of ESs (Wang et al. 2022; Weiskopf et al. 2020) can directly change regional hydrological conditions, vegetation growth, and carbon emissions (Liu et al. 2024b). For example, the main drivers of various ESs in Gannan almost include temperature or precipitation (Zhou et al. 2025). However, different from previous studies (Li et al. 2024b; Zhang et al. 2024b), this study found that the combined influence of climate factors on the spatial distribution of IESI in CYP is not significant. The possible reasons are twofold: On one hand, although the adverse effects of global climate change on ecosystems are becoming increasingly prominent, in the study area, extreme climate events occur relatively infrequently, and the heterogeneity of climate effects is less pronounced (Han et al. 2018). On the other hand, annual precipitation and evapotranspiration are the main factors influencing WY and SC, but they have a relatively smaller impact on CS and HQ (Yu et al. 2022). The changes in ESs are not solely influenced by individual factors; the interaction between population density and RDLS, as well as between LUI and RDLS, are the primary interaction variables in CYP. This indirectly suggests that LUCC has a substantial effect on the variations in CES. This highlights that through the interaction of topographic factors, the driving effect of socioeconomic factors is significantly amplified. The mountainous terrain, by restricting the intensity of development, makes the pressure exerted by economic activities on the ecosystem more concentrated. This finding is of great significance for formulating strategies tailored to local conditions to enhance the ESs in CYP.

### 4.4 Limitations and Future Work

This study quantified four key ESs based on the natural ecological environment of CYP and constructed the IESI using the PCA. Although IESI demonstrated scientific validity and effectiveness in assessing the ecosystem within the study area, some uncertainties remain in the estimation results. For instance, the CS primarily relied on previous studies, with calculations based on empirical methods or carbon density coefficients from similar regions. Therefore, future research on CS services should aim for more accurate quantification by obtaining field data. Additionally, since ESs are influenced by multiple factors, this study utilized the OPGD model to quantify their contributions. However, how to thoroughly explore the coupling effects among these factors in CYP remains a challenge for further research. Based on the assessment of CES and key driving factors in CYP, in the future, it will be feasible to combine the differences in the physical geographical environment of CYP with existing plans such as the “Master Functional Zoning Plan of Yunnan Province” and the “Development Plan for the Central Yunnan Urban Agglomeration.” ES management zones in CYP can then be demarcated, strategies for enhancing the CES in CYP can be proposed, typical construction plans can be put forward, and the implementation of these plans can be supported.

## 5 Conclusion

This study conducted a quantitative assessment of four key ESs—WY, CS, HQ, and SC—in CYP from 2000 to 2020. The IESI was constructed using the PCA, and the OPGD model was employed to explore the driving factors. Key findings include: (1) The construction of IESI using PCA provides an objective and scientific integration of various ESs and has been effectively applied in CYP. (2) Individual ESs in the study area exhibit different temporal variation characteristics. During the study period, three key ESs (WY, HQ, and SC) showed an overall declining trend, while CS displayed an increasing trend. (3) The CES in the study area first weakened and then strengthened over time. The average IESI values for CYP in 2000, 2005, 2010, 2015, and 2020 were 0.7338, 0.6981, 0.6947, 0.6650, and 0.6992, respectively, with 2015 marking a turning point. Spatially, IESI in CYP exhibits a distribution pattern of being higher in the northwest and lower in the southeast, with area transfers mainly occurring among the excellent, moderate, and good levels. (4) The 4500 m × 4500 m grid is the optimal geographic detection scale, and RDLS, slope, and NDVI are the three factors that most significantly influence the spatial differentiation of IESI. The research findings can serve as a prerequisite and basis for the overall improvement of ecological environment quality and the formulation of regional development regulations and other policies in CYP.

## Author Contributions

**Lanfang Liu:** conceptualization (equal), formal analysis (equal), writing – original draft (equal), writing – review and editing (equal). **Jinliang Wang:** funding acquisition (equal), project administration (equal), writing – review and editing (equal). **Jie Li:** conceptualization (equal), validation (equal). **Suling He:** data curation (equal), methodology (equal). **Yongcui Lan:** software (equal), visualization (equal). **Fang Liu:** methodology (equal), validation (equal).

## Conflicts of Interest

The authors declare no conflicts of interest.

## Supporting information


Appendix S1.

## Data Availability

The data that support the findings of this study are available in the main text or appendices. In addition, the data that support the findings of this study and the R code are freely downloadable from Dryad at https://doi.org/10.5061/dryad.6t1g1jx93.
