# Supplementary material for: Evaluation of Ecosystem Service Capacity Using the Integrated Ecosystem Services Index at Optimal Scale in Central Yunnan, China
Source: Ecol Evol. 2025 Apr 11;15(4):e71222. doi: 10.1002/ece3.71222 (PMC11992009; doi:10.1002/ece3.71222)
Supplement: Supplementary file 1 — Appendix S1. [file ECE3-15-e71222-s001.docx]

# Appendices

Table A1 Driver type selection and description

| Type | Driving factors | Description | Unit |
| --- | --- | --- | --- |
| Socio-economic | Artificial Nighttime-light（X1） | Characterizing the overall state of the regional economy | J/m^2^ |
|  | Land reclamation rate（X2） | Reflecting the degree of land development | % |
|  | Population density（X3） | Characterizes the distribution of resident population | Person/km^2^ |
|  | Land use intensity（X4） | Reflecting the extent of the impact of human activities on natural ecosystems | / |
| Topographic | Slope（X5） | Characterize the degree of inclination of the ground surface at the point | ° |
|  | Relief degree of land surface（X6） | Reflecting changes in regional topographic relief | m |
|  | Elevation（X7） | Reflecting regional differences in topography | m |
|  | Aspect（X8） | Characterize the orientation of terrain slopes | ° |
| Climate | Temperature（X9） | Characterize regional hot and cold conditions | ℃ |
|  | Precipitation（X10） | It is the most fundamental part of the water cycle | mm |
|  | Aridity index（X11） | Characterize the degree of wetness or dryness of the area | % |
|  | Evaporation（X12） | Characterizing regional water demand | mm |
| Vegetation | Net primary productivity（X13） | Reflects the ability of vegetation to fix atmospheric CO_2_ | gC/㎡ |
|  | Normalized Difference Vegetation Index（X14） | Characterize the state of vegetation growth in the region | / |

**Methods for assessing the amount of ESs**

**1. Water Yield**

The Water Yield module of the InVEST model was used to calculate the water yield in CYP (Huang et al., 2022; Yu et al., 2022). The Water Yield assessment module is based on Budyko's (1974) coupled hydrothermal equilibrium assumptions and average annual precipitation data; it uses the following equation:

$Y\left( x \right)=\left( 1-\frac{AET\left( x \right)}{P\left( x \right)} \right)P\left( x \right)$ (A1)

$\frac{AET\left( x \right)}{P\left( x \right)}=1+\frac{PET\left( x \right)}{P\left( x \right)}$-$\left[ 1+\left( \frac{PET\left( x \right)}{P\left( x \right)} \right)^{\omega} \right]^{1/\omega}$ (A2)

$PET\left( x \right)=K_{c}(l_{x})\times ET_{0}(l_{x})$ (A3)

$\omega\left( x \right)=\frac{Z\times AWC(x)}{P(x)}$ +1.25 (A4)

$AWC_{x}=\min\left( \max layer\_depth_{x},Root\_depth_{x} \right)\cdot PAWC$ (A5)

$$PAWC\text{ }\text{=}54.509-0.132sand-0.003(sand)^{2}-0.055silt$$

$$\text{ }-0.006(silt)^{2}-0.738clay+0.007(clay)^{2}$$

$\text{ }-2.688OM+0.501(OM)^{2}$ (A6)

where $Y\left( x \right)$ is the annual water yield of each grid cell $x$ in the study area (mm); $AET\left( x \right)$ is the annual actual evapotranspiration of grid cell $x$ (mm); $P\left( x \right)$ is the annual precipitation of grid cell $x$ (mm); $PET\left( x \right)$ is the potential evapotranspiration; $\omega(x)$ is a nonphysical parameter of natural climate-soil properties and is an empirical parameter; $ET_{0}(x)$ is the reference crop evapotranspiration for raster cell $x$; $K_{c}(l_{x})$ is the plant (vegetation) evapotranspiration coefficient for a specific land use type in raster cell $x$; and The $Z$ parameter is an ecohydrological parameter, also known as the seasonal constant, used to characterize the seasonal characteristics of regional climate, rainfall intensity, and topographic features, and it has values from 1 to 30 (Sharp et al., 2018); in this study, $Z$=6.23 was selected based on previous research (Yu et al., 2022) and experience. $AWC_{x}$ is soil available water capacity in grid $x$ (mm); $layer\_depth_{x}$ is root burial depth (mm) from HWSD; $Root\_depth_{x}$ is plant root depth (mm), which was assigned bydifferent land-use types (Table A2); $PAWC$ is the plant available water capacity (0-1); sand, silt, clay are the sand, silt and clay content of the soil (%), and OM is the organic matter content of the upper soil (%).

Table A2. Parameters of land use types in Annual Water yield module of InVEST model

| Land use type | LUCODE | lulc_veg | K_C_ | ROOT_DEPTH |
| --- | --- | --- | --- | --- |
| paddy field | 1 | 1 | 0.8 | 700 |
| irrigable land | 2 | 1 | 0.2 | 100 |
| dry field | 3 | 1 | 0.2 | 100 |
| bare land | 4 | 0 | 0.5 | 10 |
| orchard | 5 | 1 | 0.7 | 3000 |
| forest | 6 | 1 | 0.8 | 7000 |
| shrub | 7 | 1 | 0.398 | 5000 |
| grassland | 8 | 1 | 0.65 | 2500 |
| construction land | 9 | 0 | 0.3 | 500 |
| mining and storage land | 10 | 0 | 0.3 | 500 |
| highway land | 11 | 0 | 0.3 | 500 |
| water | 12 | 0 | 1 | 500 |
| rock | 13 | 0 | 0.3 | 10 |

**2. Carbon Storage**

We used the Carbon Storage and Sequestration module, which serves as a library-based method (Sharp et al., 2018) that calculates total regional carbon storage to calculate the CS in CYP. The calculation equation is as follows:

$C_{tot}=C_{above}+C_{below}+C_{soil}+C_{dead}$ (A7)

where $C_{tot}$ is the total regional CS; $C_{above}$ is the aboveground CS; $C_{below}$ is the belowground root CS; $C_{soil}$ is the soil CS; and $C_{dead}$ is the dead organic matter CS. The carbon densities for $C_{above}$ and $C_{dead}$ in CYP were determined based on previous researches (Fang et al., 2018; Liu et al., 2019; Sun, 2017), the carbon density table of CYP was compiled (Table A3).

Table A3 Table of carbon density parameters in CYP based on the InVEST model (t/hm^2^)

| LUCODE | Type | C_above_ | C_below_ | C_soil_ | C_dead_ |
| --- | --- | --- | --- | --- | --- |
| 1 | Cultivated land | 26.41 | 2.64 | 43.94 | 0 |
| 2 | Woodland | 44.75 | 12.08 | 52.71 | 1.9 |
| 3 | Grassland | 38.67 | 49.11 | 53.7 | 0.1 |
| 4 | Construction land | 18.24 | 0 | 41.61 | 0 |
| 5 | Water | 22.32 | 0 | 44.29 | 0 |
| 6 | Unutilized land | 9.49 | 0 | 50.77 | 0 |

**3. Habitat Quality**

The Habitat Quality module of the InVEST model was used to calculate the habitat quality in CYP. The model obtains habitat distribution characteristics by establishing the relationship between different land use types and threat sources (Chen et al., 2016; Sharp et al., 2018). In this paper, we used paddy fields, drylands, and construction land as habitat stressors and referred to previous studies (Huang et al., 2018; Yang et al., 2021) to assign values to the maximum impact distance of stressors, weights, suitability of each habitat type and sensitivity of habitats to stressors (Tables A4 and A5), calculated as follows:

$Q_{xj}=H_{j}\left( 1-\left( \frac{D_{xj}^{z}}{D_{xj}^{z}+K^{z}} \right) \right)$ (A8)

$D_{xj}=\underset{r=1}{\overset{R}{\sum}}\underset{y=1}{\overset{Y_{r}}{\sum}}\left( \frac{W_{r}}{\sum_{r=1}^{R} W_{r}} \right)r_{y}i_{rxy}\beta_{x}S_{jr}$ (A9)

where $Q_{xj}$ is the habitat quality of raster $x$ in LULC type $j$; $D_{xj}$ is the total threat level of raster $x$in LULC or habitat type $j$; K and Z are scaling factors; $H_{j}$ is the habitat suitability of LULC type $j$; $R$ denotes the stress factor; $Y_{r}$ is the number of grids occupied by stressor $r$; $W_{r}$ indicates the weight of the stressor (WEIGHT), with values from 0 to 1; $r_{y}$ is the stressor value of grid $y$; $i_{rxy}$denotes the stress level of stress factor value $r_{y}$ of raster $y$ to habitat raster $x$; $\beta_{x}$ denotes the accessibility level of raster $x$; and $S_{jr}$ denotes the sensitivity of habitat type $j$ to stress factor $r$.

Table A4 Threat factors and their maximum influence distance, weight and type of decay

| THREAT | MAX_DIST | WEIGHT | DECAY |
| --- | --- | --- | --- |
| Paddy field | 5 | 0.7 | Exponential |
| Dry field | 8 | 0.8 | Exponential |
| Construction land | 10 | 0.9 | Linear |

Table A5 Sensitivity of different land use types to different ecological threat factors

| LUCODE | NAME | HABITAT | Paddy field | Dry field | Construction land |  |
| --- | --- | --- | --- | --- | --- | --- |
| 1 | paddy field | 0.3 | 0 | 0 | 0 |  |
| 2 | irrigable land | 0.25 | 0 | 1 | 0 |  |
| 3 | dry field | 0.25 | 0 | 0 | 0 |  |
| 4 | bare land | 0 | 0.2 | 0.2 | 0.3 |  |
| 5 | orchard | 0.6 | 0.5 | 0.5 | 0.5 |  |
| 6 | forest | 0.9 | 0.5 | 0.7 | 0.6 |  |
| 7 | shrub | 0.6 | 0.3 | 0.6 | 0.6 |  |
| 8 | grassland | 0.7 | 0.5 | 0.5 | 0.6 |  |
| 9 | construction land | 0 | 0 | 0 | 0 |  |
| 10 | mining and storage land | 0 | 0 | 0 | 0 |  |
| 11 | highway land | 0 | 0 | 0 | 0 | |
| 12 | water | 0.8 | 0.7 | 0.8 | 0.6 | |
| 13 | rock | 0 | 0.2 | 0.2 | 0.3 | |

**4. Soil Conservation**

In this paper, the revised soil erosion model RUSLE (Revised Universal Soil Loss Equation) (Renard et al.,1997) was used to estimate the soil retention in CYP. The calculation equation is as follows:

$A=R\times K\times LS\times(1-C\times P)$ (A10)

where $A$ is the soil conservation amount per unit area, i.e., the difference between potential soil erosion and actual soil erosion, also known as reduced soil erosion per unit area (t/(hm^2^-a)); $R$ is the rainfall erosion force factor (MJ·mm/(hm^2^·h·a)); $K$ is the soil erodibility factor (t·hm^2^·h/(MJ·hm^2^·mm)); $LS$ is the slope gradient and slope length factor (dimensionless); $C$ is the vegetation cover and management factor (dimensionless); and $P$ is the soil and water conservation measure factor (dimensionless).

(1) Rainfall erosion force (R)

The rainfall erosion force is the potential capacity of rainfall to cause soil erosion, which is the main driving factor leading to soil erosion (Fang et al., 2015). Based on available monthly meteorological data information and model applicability in CYP, the final calculation method used in this study is shown in Eq. (S11). (Silva, 2004):

$R=\underset{i=1}{\overset{12}{\sum}}73.989\times\left( \frac{P_{i}^{2}}{P_{a}} \right)^{0.7387}$ (A11)

where $R$ is the annual rainfall erosion force (MJ·mm)/(hm^2^·h·a); $P_{i}$ is the rainfall of the ith month (mm); and $P_{a}$ is the annual average rainfall (mm). The data required by this method are easy to obtain and have high accuracy in areas with complex terrain.

(2) Soil erosion force (K)

Soil erodibility refers to the ease of soil particles being hydraulically separated and transported (Li et al., 2018), and soil erodibility is mainly related to soil physicochemical properties such as soil texture, organic matter content, soil structure, and permeability. The productivity model Erosion Productivity Impact Calculator (EPIC) was used to quantify the soil erodibility K factor, and the results were revised (Chen et al., 2018; Williams et al., 1983) to make them more consistent with the soil properties in China.

The EPIC model used to calculate the soil erodibility factor (K) is given by:

$K=\left（ -0.01383+0.51575K_{EPIC} \right）*0.1317$ (A12)

$$K_{EPIC}=\left\{ 0.2+0.3exp\left[ 0.0256SAN\left( 1-\frac{SIL}{100} \right) \right] \right\}\times\left( \frac{SIL}{CLA+SIL} \right)^{0.3}\times$$

$\left( 1-\frac{0.25C}{C+exp(3.72-2.95C} \right)\times\left( 1-\frac{0.7（1-SAN）}{（1-SAN）+exp(22.9（1-SAN）-5.51)} \right)$ (A13)

where SAN is the sand content (%), SIL is the silt content (%), CLA is the clay content (%), and C is the organic carbon content (%).

(3) Slope gradient and slope length factor (LS)

The LS factor reflects the influence of topography on soil erosion (Hu et al., 2020). In this study, the equation proposed by Wischmeier & Smith (1965) and McCool (1989) was used to calculate the slope length L-factor in CYP:

$L=\left( \frac{\lambda}{22.13} \right)^{\alpha}$ (A14)

$\alpha=\beta/\left( 1+\beta\right)$ (A15)

$\beta=\left( sin\theta/0.089 \right)/\left( 3.0{sin}^{0.8}\theta+0.56 \right)$ (A16)

where $L$ is the slope length factor; $\lambda$ is the slope length; 22.13 is the standard plot slope length (m); $\alpha$ and $\beta$ are the slope length factor indices; and $\theta$ is the slope degree.

For the geomorphological characteristics of the study area, the slope S-factor in CYP was calculated by segmentation, combining the results of McCool (1989) and Liu (2000) and other scholars, and the formula is as follows:

$S=\left\{ \begin{aligned} &10.8sin\theta+0.03,\theta<5^{\circ} \\ &16.8sin\theta-0.05,5^{\circ}\leq\theta<10^{\circ} \\ &21.92sin\theta-0.96,\theta\geq10^{\circ} \end{aligned} \right.$ (A17)

where $S$ is the slope factor, and $\theta$ is the slope.

By multiplying the slope length L factor and slope S factor, we obtained the topographic LS factor in CYP.

(4) Vegetation cover and management factor (C)

The vegetation cover and management factor indicate the influence of vegetation cover and management measures on soil erosion, and the value is closely related to vegetation cover and takes values in the range of [0,1]. In this paper, the C-value calculation method proposed by Cai et al. (2000) was used. The calculation formula is as follows:

$C=\left\{ \begin{aligned} &1，0\leq F_{c}<0.096 \\ &0.6508-0.3436\lg\left( F_{c} \right),0.096\leq F_{c}\leq78.3 \\ &0,F_{c}>78.3 \end{aligned} \right.$ (A18)

where $C$ is the vegetation cover management factor, and $F_{c}$ is the vegetation coverage (%).

(4) Soil and water conservation measures factor (P)

The P-factor refers to the ratio of soil erosion under certain soil and water conservation measures to that under downhill planting, with values ranging from 0 to 1, where a value of 0 means no soil erosion will occur and a value of 1 means no soil and water conservation measures are taken (Lu et al., 2017). Based on the survey of the current situation of soil and water conservation in CYP, each feature type was assigned a corresponding P value based on the table of P values for different soil and water conservation engineering measures in China (Wang and Jiao, 1996) (Table A6).

Table A6 Soil and water conservation measures factor P value

| Type | Paddy field | Dry field | Woodland | Grassland | Water | Construction land | Unutilized land |
| --- | --- | --- | --- | --- | --- | --- | --- |
| P Value | 0.01 | slope<5,0.11;  5≦slope<10,0.22;  10≤slope<15,0.31;  15≤slope<20,0.58;  20≤slope<25,0.71;  slope≥25,0.8 | 1 | 1 | 0 | 0 | 1 |

**Reference**

Budyko (1974). *Climate and Life.* Academic Press, New York.

Cai, C., Ding, S., Shi, Z., Huang, L., &Zhang, G., (2000). Study of Applying USLE and Geographical Information System IDRISI to Predict Soil Erosion in Small Watershed. *Journal of Soil and Water Conservation* 19–24. https://doi.org/10.13870/j.cnki.stbcxb.2000.02.005

Chen, B., Qu, J., Ge, M., Shen, Y., Wang, A., &Wang, G., (2018). Spatio-temporal Analysis on Soil Erosion over Xuzhou City. *Journal of Geo-information Science 20*, 1622–1630.

Chen, Y., Qiao, F., &Jiang, L., (2016). Effects of Land Use Pattern Change on Regional Scale Habitat Quality Based on InVEST Model—a Case Study in Beijing. *Acta Scientiarum Naturalium Universitatis Pekinensis 52*, 553–562. https://doi.org/10.13209/j.0479-8023.2016.057

Fang, G., Xiang, B., Zhao, W., Xie, Q., Diao, Z., &Chi, W., (2015). Study on Soil Erosion in LaSa River Basin Based on GIS and RUSLE. *Journal of Soil and Water Conservation 29*, 6–12. https://doi.org/10.13870/j.cnki.stbcxb.2015.03.002

Fang, J., Yu, G., Liu, L., Hu, S., &Chapin, F.S., (2018). Climate change, human impacts, and carbon sequestration in China. *The Proceedings of the National Academy of Sciences* *115*, 4015–4020. https://doi.org/10.1073/pnas.1700304115

Hu, X., Li, Z., Chen, J., &Nie, X., (2020). Soil Conservation Benefits of the Grain for Green Program in the Hilly Red Soil Region of Southern China. *Journal of Soil and Water Conservation 34*, 95–100. <https://doi.org/10.13870/j.cnki.stbcxb.2020.06.015>

Huang, X., Peng, S., Wang, Z., Huang, B., & Liu, J., (2022). Spatial heterogeneity and driving factors of ecosystem water yield service in Yunnan Province，China based on Geodetector. *Chinese Journal of Applied Ecology* *33*, 2813–2821. https://doi.org/10.13287/j.1001-9332.202210.025

Huang, X., Yang, Y., Wu, Y., Gao, Y., Gu, Y., &Yuan, Z., (2018). Land Use Change and Its Impact on Habitat Quality in Karst Nature Reserve from 1990 to 2017. *Bulletin of Soil and Water Conservation 38*, 345–351. https://doi.org/10.13961/j.cnki.stbctb.2018.06.052

Li, M., Fang, R., Le, F., Wu, F., &Zhang, X., (2018). Quantitative Monitoring of Soil and Water Loss in Key Control and Prevention Areas of Jiangsu Province Based on RS and GIS. *Bulletin of Soil and Water Conservation 38*, 228–233. https://doi.org/10.13961/j.cnki.stbctb.2018.04.037

Liu, B., Nearing, M.A, Shi, P., Jia, Z., (2000). Slope Length Effects on Soil Loss for Steep Slopes. *Soil Science Society of America Journal. 64,* 1759–1763. https://doi.org/10.2136/sssaj2000.6451759x

Liu, X., Li, X., Liang, X., Shi, H., &Ou, J., (2019). Simulating the Change of Terrestrial Carbon Storage in China Based on the FLUS-InVEST Model. *Tropical Geography 39*, 397–409. https://doi.org/10.13284/j.cnki.rddl.003138

Lu, C., Dai, F., &Liu, G., (2017). Spatial Distribution Characteristics of Soil Conservation Service in Wanzhou District Based on GIS And RUSLE Model. *Resources and Environment in the Yangtze Basin* 26, 1228–1236.

McCool, D.K., Foster, G.R., Mutchler, C.K., &Meyer, L.D., (1989). Revised Slope Length Factor for the Universal Soil Loss Equation. *Transactions of the ASAE 32,* 1571–1576. https://doi.org/10.13031/2013.31192

Renard, K. G. , Foster, G. R. , Weesies, G. A. , Mccool, D. K. , & Yoder, D. C. . (1997). Predicting soil erosion by water: a guide to conservation planning with the revised universal soil loss equation (rusle).*Agricultural Handbook.*

Sharp, R., Chaplin-Kramer, R., Wood, S., Guerry, A., Tallis, H., Ricketts, T., Nelson, E., Ennaanay, D., Wolny, S., Olwero, N., Vigerstol, K., Pennington, D., Mendoza, G., Aukema, J., Foster, J., Forrest, J., Cameron, D. R., Arkema, K., Lonsdorf, E., & Douglass, J. (2018). *InVEST User’s Guide*. https://doi.org/10.13140/RG.2.2.32693.78567

Silva, A.M. da, 2004. Rainfall erosivity map for Brazil. *CATENA 57*, 251–259. https://doi.org/10.1016/j.catena.2003.11.006

Sun, X., (2017). *Evaluation of ecosystem services in Shangri-La based on InVEST model* (master’s degree). Yunnan Normal University.

Wang, W., &Jiao, J., (1996). Quantitative Evaluation on Factors Influencing Soil Erosion in China. *Bulletin of Soil and Water Conservation* 1–20.

Williams, J.R., Renard, K.G., &Dyke, P.T., (1983). EPIC: A new method for assessing erosion’s effect on soil productivity. *Journal of Soil and Water Conservation 38*, 381–383.

Wischmeier, W.H. and Smith, D.D. (1965) *Predicting rainfall-erosion losses from cropland east of the Rocky Mountains: guide for selection of practices for soil and water conservation*, Agricultural Research Service, U.S. Dept of Agriculture in cooperation with Purdue Agricultural Experiment Station

Yang, W., Li, S., Peng, S., Li, Y., Zhao, S., &Qiu, L., (2021). Identification of important biodiversity areas by InVEST model considering opographic relief: A case study of Yunnan Province, China. *Chinese Journal of Applied Ecology 32*, 4339–4348. https://doi.org/10.13287/j.1001-9332.202112.004

Yu, Y., Sun, X., Wang, J., & Zhang, J., (2022). Using InVEST to evaluate water yield services in Shangri-La, Northwestern Yunnan, China. *PeerJ* *10*, e12804. <https://doi.org/10.7717/peerj.12804>
